# Innovative sensors with selectivity enhancement by molecularly imprinted polymers for the concurrent quantification of donepezil and memantine†

**DOI:** 10.1039/d5ra02850g

**Published:** 2025-06-03

**Authors:** Eman M. Moaaz, Ahmed S. Fayed, Ezzat M. Abdel-Moety, Mamdouh R. Rezk

**Affiliations:** a Pharmaceutical Analytical Chemistry Department, Faculty of Pharmacy, Cairo University Kasr El-Aini Street ET-11562 Cairo Egypt eman.moaaz@pharma.cu.edu.eg

## Abstract

Ion-selective sensors are widely employed in various pharmaceutical, environmental, and biological analytical applications due to their simplicity, cost-effectiveness, and rapid response times. They suffer from some challenges though. These challenges may arise from the selective sensing process that can be hindered by interference from ions with similar charges or suitable lipophilicity. Solid contact type due to water layer formation between the sensing surfaces may also appear as an obstacle. This work is dedicated to overcoming the selectivity issue using the molecularly imprinted polymer (MIP) approach to determine Donepezil (DON) and Memantine (MEM) in their combined pharmaceutical formulation. Precipitation polymerization approach was employed for the preparation of the MIP for each drug. The resulting MIPs were thoroughly examined using various characterization methods. The potential response of the proposed sensors was stabilized by applying graphene nanoplatelets as an ion-to-electron transducer layer. This layer prevented the formation of the water layer, improved the responses, and enhanced charge transfer. Two sensors featuring different cationic exchangers were designed for the selective determination of donepezil, for which one sensor was developed for memantine analysis by adding the corresponding MIPs to the membrane components. The achieved detection limits were 5.01 × 10^−8^ M & 4.47 × 10^−7^ M for DON and 2.24 × 10^−7^ M for MEM, with slope values of 56.77 mV per decade, 56.91 mV per decade, and 55.87 mV per decade, respectively. Each sensor was successfully employed for the selective determination of its corresponding drug in the combined formulations and spiked human plasma samples without interference.

## Abstract 2

This work is overcoming the selectivity issue using the molecularly imprinted polymer (MIP) approach to determine donepezil (DON) and memantine (MEM) in their combined pharmaceutical formulation.

## Graphical abstract

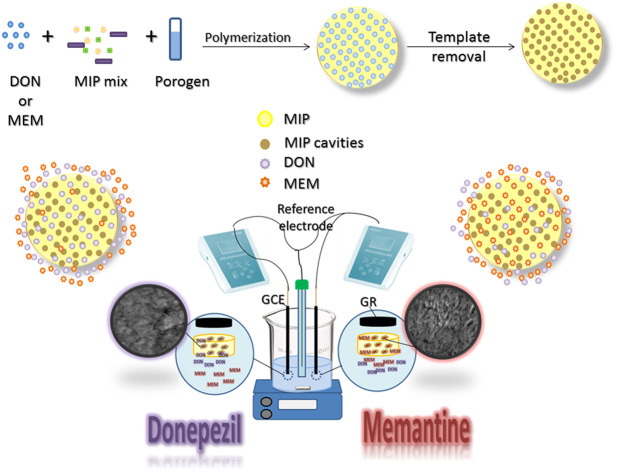

## Introduction

Potentiometric ion selective electrodes (ISEs) have been widely used in pharmaceutical drug analysis. They have the advantages of being simple, rapid, of low energy consumption, and low sample concentrations.^1,2^ Solid contact ion-selective electrodes (SC-ISEs) can offer rigid portable sensors that produce robust responses. However, SC-ISEs suffer from irregular potential drift, probably due to water layer formation between the ion-selective membrane (ISM) and the sensor surface. This layer serves as an electrolyte storage that re-equilibrates upon changing the concentration of the analyzed samples, affecting the stability and sensitivity of the electrodes.^3,4^ To conquer this obstacle, various hydrophobic transducer interlayers, such as graphene (GR), were investigated.^5–8^ GR is a layer or multiple layers of graphite with excellent electrochemical^9,10^ and electronic^11^ properties and high chemical stability.^10^ It has been used widely in establishing electrochemical sensors and biosensors^6^ as an excellent hydrophobic transducer material. GR-based nanoparticles and nanoplatelets have been effectively utilized as transducer layer in SC-ISEs enhancing their stability and sensitivity.^2,6,12–15^

The functionality of the ISM depends on the presence of an ion exchanger where its counter ion is replaced by the target analyte in the ionic form. Consequently, the exchangers are generally classified as either cationic for positively charged analytes or anionic for negatively charged ones. However, the presence of other ions with the same charge as the target analyte in the sample, especially with an optimum degree of lipophilicity, will interfere with the proper sensing of the main ion. This challenge necessitates incorporating selectivity-enhancing carrier substances such as ionophores and specially fabricated molecularly imprinted polymers (MIPs).^16^

MIPs are polymers imprinted by a specific interaction between the functional monomers containing acidic or basic groups and the target drug as a template, usually in the presence of a crosslinking monomer and an initiator.^17^ After the non-covalent copolymerization process, the template molecules are detached, leaving recognition micro-cavities matching the template in shape, size, and functional groups' arrangement. The high cross-linker ratio maintains the shape memory of these cavities after template removal, keeping the functional groups in the suitable arrangement for recognizing and rebinding to the target molecules.^18^ The interaction between the imprinted recognition sites and the target analytes resembles that of antigen–antibody reversible interactions such as H-bonds, ionic-bonds, hydrophobic interactions, and van der Waals forces.^17^ Therefore, MIPs are known as synthetic receptors that are designated to be of relatively low cost, straightforward synthesis with multiple approaches, chemically and thermally stable, easy to handle, and reusable without activity loss.^19^ These advantages over biological materials encourage the use of MIPs in different applications: separation methods,^20^ electrochemical techniques,^21–23^ and biological and food analysis.^17,24^

Donepezil hydrochloride (DON) and memantine hydrochloride (MEM) are central nervous system active drugs that are prescribed in combination for controlling Alzheimer's disease (AD). Where, DON is an acetylcholinesterase inhibitor that limits nerve deterioration and elaborates cholinergic stimulation,^25^ and MEM is *N*-methyl-d-aspartate receptor antagonist that improves cognitive symptoms.^26^ The combination of both drugs helps in the management of the progressive stages of AD.^27,28^ The literature survey revealed that the combination was analyzed by some chromatographic methods.^29–34^ When, only an ISE-method was published for DON-analysis,^35^ and many other ISE-methods for MEM,^36–39^ including one that applied the MIP-approach in the sensing process.^40^

The current study is directed to use the MIP approach for the concurrent analysis of DON and MEM. Firstly, the selection of the cationic exchanger was investigated for each drug, then three sustainable GR-modified glassy carbon electrodes (GCE) were prepared with the incorporation of the MIPs. Two sensors with different cationic exchangers were used for the selective determination of DON and one for the selective analysis of MEM. The selectivity of each sensor was evaluated by the opposite co-formulated drug and compared to MIP-free sensors. The proposed sensors were applied to determine both drugs in their combined pharmaceutical formulation and spiked human plasma. The sustainability of the proposed potentiometric method was assessed by the three metric tools, namely; Analytical GREEnness metric tool (AGREE),^41^ White Analytical Chemistry (WAC) approach,^42^ and Modified Green Analytical Procedure Index (MoGAPI).^43^

## Experimental

### Material and methods

#### Chemicals and reagents

DON (B.N.: DH21080018) was kindly provided by Apex Pharma (Cairo, Egypt), while MEM (B.N.: RI22-194) was generously provided by Marcyrl Pharmaceutical Ind. (Cairo, Egypt). Their potencies were investigated by HPLC-methods,^44,45^ yielding results of 100.01% ± 1.45% for DON & 99.95% ± 1.8% for MEM. Two versions of Mixmazil® capsules, each labeled to contain 10.0 mg DON dispensed either with 14 mg or 28 mg MEM (BN: 001, for both formulations) per capsule, were friendly obtained from Hikma Pharmaceutical Company (Giza, Egypt). All used chemicals were of high analytical grade.

Azobisisobutyronitrile (AIBN), calix[6]arene (CX6), dimethylsulfoxide (DMSO), ethylene glycol dimthacrylate (EGDMA), ethanol, methanol, glacial acetic acid, methacrylic acid (MAA), polyvinyl chloride (PVC), potassium tetrakis (*p*-chlorophenyl) borate (K-TCPB), potassium tetrakis [3,5 bis(trifluoromethyl)phenyl] borate (K-TFMPB), sodium tetraphenylborate (TPB), phosphomolybdic acid (PMA), 2-nitrophenyl octyl ether (NPOE), tetrahydrofuran (THF), potassium chloride (KCl), sodium chloride (NaCl), potassium ferrocyanide (K_4_[Fe(CN)_6_]), and potassium ferricyanide (K_3_[Fe(CN)_6_]), were purchased from Sigma Aldrich (MO, USA). Di-sodium hydrogen phosphate was purchased from E. Merck (Darmstadt, Germany). *o*-phosphoric acid was from SD Fine-Chem. Ltd Company (Mumbai, India). Graphene nanoplatelets (6–8 nm thick × 5 microns wide) were obtained from Strem Chemicals INC. (Newburyport, USA). Ultra-pure HPLC-grade water was purified by New Human Power 1 device (Human Corporation, Seoul, Korea). Human plasma was purchased from the Holding Company for Biological Products and Vaccines (VACSERA, Cairo, Egypt).

#### Instruments

A Jenway digital ion analyzer model 3540 (Essex, UK) coupled with Ag/AgCl double-junction reference electrode (Thermo Scientific Orion 900200, MA, USA); 3.0 M KCl is the inner filling solution and 10% KNO_3_ is used as a bridge electrolyte) to measure the potential difference. A Jenway pH glass electrode (Essex, UK) was used for pH adjustments. Glassy carbon electrodes (GCE) (OD:10 mm, ID, 5 mm) CH instruments (Austin, USA) as working electrodes. Scanning electron microscope (SEM) FEG model Quanta 250 (Fei Company, Oregon, USA). Brunauere-Emmette-Teller (BET) analyzer BELSORP model Microtrac (Osaka, Japan). IR spectrometer model 1310 PerkinElmer (Norwalk, USA). Sigma centrifuge device (Focus Scientific, Ireland). Potentiostat/Galvanostat Metrohm Autolab PGSTAT204 (Utrecht, Netherland) operated with Nova 1.11 software has been utilized to perform electrochemical impedance measurements. The dynamic size of graphene nanoplatelets was measured using a Zetasizer (Malvern INS., UK) *via* dynamic light scattering technique (DLS).

### Synthesis of MIPs for DON and MEM

The MIPs were prepared *via* precipitation polymerization method.^23,46^ The molar ratios used in the preparation of MIPs were selected based on previously reported successful protocols.^21,40,47^ Each MIP was prepared in a 50-mL glass-capped bottle using 0.5 mmol of each drug as a template dissolved in 40.0 mL of DMSO as a porogenic solvent, then 2.0 mmol of MAA functional monomer was added and sonicated for 15.0 min. After that, 8.0 mmol of cross-linker EGDMA and 0.6 mmol of AIBN initiator were added and sonicated for 1.0 min. The mixtures were purged with nitrogen gas for 15.0 min and placed in a thermostatic oil bath at 60 °C for 24.0 h. The formed precipitates were filtered by decantation to get rid of excess solvent. They were then washed with ethanol and centrifuged several times to remove unreacted materials. To leach the templates from the imprinted polymers, they were washed with a solvent mixture of methanol and acetic acid (9 : 1, v/v) several times. DON template removal was confirmed by the absence of the drug peaks in the effluent by using UV/vis spectroscopic analysis. However, it was difficult to monitor MEM extraction at 190.0 nm, the maximum wavelength of MEM. Thus, the same washing steps for DON removal were performed for MEM removal, followed by spotting the last wash on a thin layer of aluminum plate and dipping the plate in Dragendorff's solution. The absence of a darker color in the spotting place confirmed the removal of the MEM template. Finally, the obtained MIPs were rinsed with distilled water several times till neutral pH was achieved, then dried at 100 °C for 2 hours. The non-imprinted polymer (NIP) was prepared as mentioned for the MIPs, except for the addition of the template.

### Preparation of graphene nanoplatelets (GR) transducer

Using the solution dispersion technique,^48^ 10 mg of graphene nanoplatelets were disseminated into 1 mL THF and ultra-sonicated for 5 minutes.

### Size distribution measurements of graphene nanoplatelets

Graphene nanoplatelets were dispersed by weighting 1.5 mg into 20 mL methanol and sonicated for 30 minutes. From this dispersion stock, 1 mL was further diluted with 6 mL methanol and sonicated for another 15 minutes prior to DLS analysis.

### Preparation of ion-selective electrodes (ISEs)

The ISM components were prepared in screw-cap tubes by mixing 95 mg of PVC, 10 mg of functional polymer (either MIP for each drug or NIP), 5 mg of the cationic exchanger (TPB, K-TCPB, K-TFMPB, or PMA), and 0.2 mL of NPOE, then all dissolved in 3 mL THF. The surface of the working GCE was polished sequentially with alumina slurry of 1.0, 0.3, and 0.05 μm particle sizes, followed by rinsing and sonication to remove remaining particles in purified water.

The transducer layer was formed by drop-casting 10 μL of GR-nanoplatelets on the electrode surface, allowing it to dry completely. Subsequently, 20 μL of the ISM was drop-casted on the top of the transducer layer, left to dry, then conditioned overnight in 1 × 10^−4^ M of the target drug solution. Furthermore, more sensors were prepared by omitting either the polymers, GR, or both to elect the optimal exchanger and for comparison purposes.

### Standard stock and working solutions preparations

The stock solution for each drug was prepared in 20 mL phosphate buffer pH 5.5 ± 0.1 at a concentration of 1 × 10^−2^ M. The working solutions of descending concentrations (1 × 10^−3^ M to 1 × 10^−8^ M) for DON and (1 × 10^−3^ M to 1 × 10^−7^ M) for MEM were prepared by serial dilutions from stock solutions. As mentioned, the studied interfering ions solutions were prepared to get final concentrations of 1 × 10^−4^ M.

### Application to pharmaceutical formulation

Ten capsules from each strength of Mixmazil® were weighed and then emptied. Individually, the contents and void capsules were re-weighted. The granules were thoroughly combined and powdered, then amounts corresponding to 10.4 mg of DON and 14.56 mg or 29.12 mg of MEM were sonicated with 15 mL of the buffer for 30 minutes. The volumes were completed to 25 mL with the buffer to obtain final stock samples concentrations of 1.0 × 10^−3^ M for DON and, 2.7 × 10^−3^ M & 5.4 × 10^−3^ M for MEM.

### Application to spiked human plasma

One of the advantages of ISE methods is the ability to perform direct measurements of the samples without prior treatment, such as filtration or plasma protein precipitation. However, DON is 96% bound to plasma protein;^49,50^ this percentage may affect the reliable analysis of the drug. Therefore, the drug was extracted and the proteins were precipitated with methanol. A volume of 1 mL of human plasma was spiked with 2.5 mL of 1 × 10^−3^ M of each drug and sonicated for 20 minutes. Then methanol (4 mL) was added for plasma protein precipitation and drug extraction, followed by sonication for an extra 20 min. and centrifugation at 4000 rpm for 20 minutes. The supernatant was quantitatively transferred to a 25-mL volumetric flask and left in a fume hood for 1 hour to enable methanol evaporation; the volume was completed with phosphate buffer pH 5.5 ± 0.1.

### Electrochemical impedance spectroscopy (EIS) measurements

EIS measurements were conducted in a standard three-electrode setup consisting of the GCE as a working electrode, an Ag/AgCl reference electrode, and a Pt counter electrode. The experiments were carried out in a solution of 0.1 M KCl and 0.01 M K_4_[Fe(CN)_6_] & K_3_[Fe(CN)_6_]. The frequency sweep ranged from 100 kHz to 0.1 Hz with an alternating voltage amplitude of 5 mV.

## Results and discussion

Electrochemical analyses are known to be simple, expeditious, sample-preserving, and almost produce no waste.^1,2,51–53^ Particularly, ISEs require no sample preparation, making the process more efficient and maintaining the samples in their original form.^1,2,54,55^ Although of being selective, they are not entirely specific, mainly in the presence of interfering ions that carry the same charges and exhibit similar properties.^56^ In this study, the co-formulated drugs, DON and MEM, have basic nitrogen with close p*K*_a_ values of 8.9 and 10.27 and similar lipophilicity expressed by log *P* of 4.7 and 3.2 for DON and MEM, respectively.^49,50^ Therefore, both act as monovalent cations at acidic to slightly basic media and would induce significant interference that limits their simultaneous potentiometric analysis. Accordingly, their compatible MIPs were integrated to enhance the specific recognition of each drug and reduce the interference. While MIPs are designed to provide high selectivity for the target analytes, they are not flawless. The imprinting process is not always perfect; this can result in less specific binding sites, allowing some degree of interference, especially from the compounds that have similar shape, size, or functional groups to the target analyte.^17,46,57^

### Selection of suitable cationic exchangers

Four cationic exchangers on bare GCE were tested to enhance the selectivity for DON and MEM. The exchangers that exhibited better interaction (potential, sensitivity, and slope) were utilized to compensate for possible non-specific binding with MIPs and allow for more precise differentiation between DON and MEM in the mixture.

**Fig. 1:**
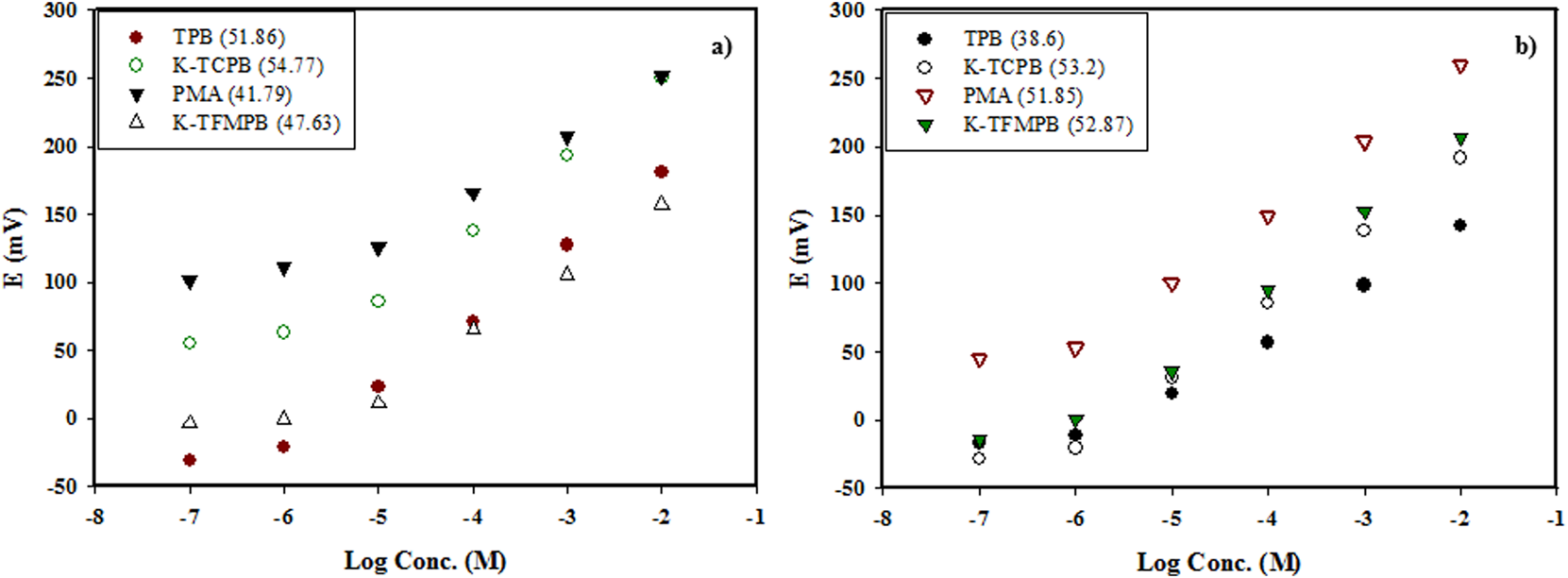
(a) DON cationic exchangers (linearity ranges (1.0 × 10^−5^ to 1.0 × 10^−2^ M) except for TPB (1.0 × 10^−6^ to 1.0 × 10^−2^ M)), b) MEM cationic exchangers [linearity ranges (1.0 × 10^−6^ to 1.0 × 10^−2^ M)].

The best responses were recorded while using TPB and K-TCPB for DON and with PMA and K-TFMPB for MEM [Fig. 1]. For DON, TPB exhibited the highest sensitivity (down to 1 × 10^−6^ M) with optimum slope. On the other hand, K-TCPB combined the advantages of high potential values and slope [Fig. 1a]. Although PMA displayed the highest potential values, the slope response was unsatisfactory. It was proposed that the interaction of DON was favorable with TPB then K-TCPB owing to low steric hindrance of the exchangers combined with optimum lipophilicity. For MEM, the benefits of high potential values and slope were presented while using PMA and K-TFMPB [Fig. 1b]. The proposed interaction of MEM with PMA and K-TFMPB was dependent on a combination of electronegativity and steric hindrance. These variations in responses between the studied drugs and cationic exchangers suggested a more efficient association between the ions of each drug and the mentioned exchangers, likely due to compatibility with their molecular structures in terms of favorable interaction, steric hindrance, in addition to mutual lipophilicity. Consequently, TPB and K-TCPB were selected for further analysis of DON, while PMA and K-TMFPB were chosen for subsequent analysis of MEM in the presence of GR as a transducer layer and their corresponding MIPs. PMA was later excluded owing to the resulting low value of the selectivity coefficient (−1.17) even in the presence of MEM-MIP compared with K-TMFPB result (−2.26) using DON as an interfering ion.

### Preparation and characterization of the MIPs

The incorporation of MIPs into ISMs required the use of a polymerization method with a high yield such as precipitation polymerization. Precipitation polymerization produces imprinted polymers in spheres with uniform size and high surface area.^18^ MAA offers a potent and selective interaction with templates, in addition to its acidic nature, which is compatible with the basic amine in the studied drugs. Therefore, it was used as the functional monomer. EGDMA was selected as the cross-linker for its ability to form polymers with rigid and porous structures. The cross-linker ratio should be high to maintain the stability and structure of the recognition sites. The ratio of 1 template: 4 monomer: 16 cross-linker was applied for MIPs synthesis.^21,57^

#### Scanning electron microscope (SEM) examination

**Fig. 2:**
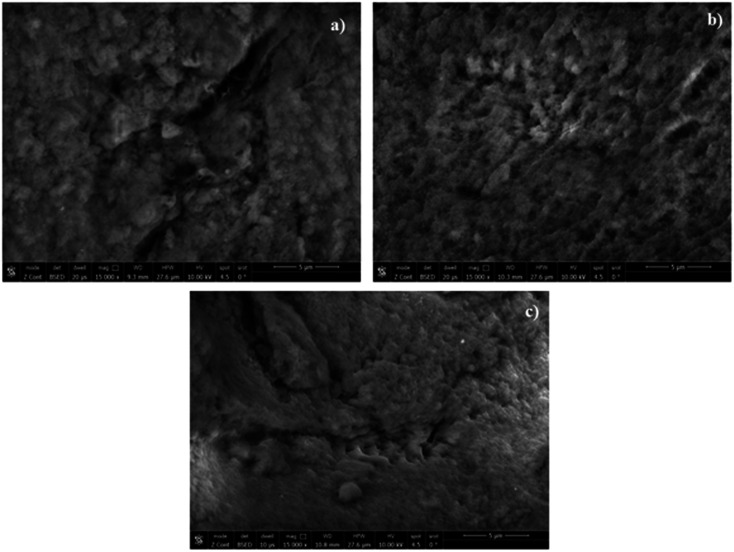
SEM surface images of: (a) DON-MIP, (b) MEM-MIP, (c) NIP.

The leached MIPs of DON and MEM, and their NIP were subjected to surface examination by SEM [Fig. 2]. The images displayed that the MIPs surfaces exhibited a more rough and porous appearance compared with the surface of the NIP. This spongy porous shape correlates to the imprinted cavities of the leached templates.^18,23^ Although the morphological differences between MIPs and NIP may not be sharply distinguished in the SEM images, the observed surface irregularities and porosity are consistent with successful imprinting. These findings are further supported by BET and binding capacity discussed in later sections.

#### Fourier-transform infrared (FT-IR)

The functional groups of the drugs, their matching leached and un-leached MIPs, and the NIP were characterized with FT-IR [Fig. S1†]. The IR spectrum of DON showed a band at 2461 cm^−1^ and 2418 cm^−1^ corresponding to the presence of protonated amine hydrochloride salts (N^+^–H). Aryl and alkyl C–H stretching at 2968 cm^−1^ and 2924 cm^−1^, in addition to C=O and cyclic C=C stretching at 1697 cm^−1^ and 1589 cm^−1^, respectively. It also showed C–O alkyl aryl ether stretching at 1292 cm^−1^ and 1266 cm^−1^. These bands were absent in the leached DON-MIP's IR spectrum; however, new bands related to the polymer components appeared. A broad band of carboxylic acid O–H stretching corresponding to MAA appeared at 3448 cm^−1^ to 3618 cm^−1^, weak vinyl C–H stretching appeared at 2989 cm^−1^ and 2988 cm^−1^ related to EGDMA and MAA, in addition to C=O band and very weak band of vinyl C=C confirming the polymer formation at 1732 cm^−1^ and 1639 cm^−1^, respectively. The IR spectrum of the un-leached DON-MIP displayed most of DON functional groups bands and the bands related to the polymer in the same places or slightly shifted due to bonds formation. However, it was observed that the band of amine hydrochloride salts (N^+^–H) significantly decreased, suggesting that successive hydrogen bonding with MAA acidic group in the polymer.

Likewise, the IR spectrum of MEM displayed an overlapping big band from 3209 cm^−1^ to 2788 cm^−1^ related to N–H stretching of primary amine and alkyl C–H stretching. It also showed small bands from 2611 cm^−1^ to 2522 cm^−1^ of the primary amine HCl salt. Amine C–N stretching and N–H bending vibration bands appeared at 1307 cm^−1^ and 1512 cm^−1^, respectively. The spectrum of the leached MEM-MIP confirmed the efficient elimination of MEM-template owing to the absence of MEM characteristic bands. The un-leached MEM-MIP's spectrum also showed the bands corresponding to the polymer structure along with MEM functional groups. A remarkable decrease in the overlapped band appearing in MEM's spectrum was observed, suggesting the binding of the amine group to MAA as well. The spectrum of the NIP showed almost the same characteristic band of the polymer that appeared in the leached MIPs with a slightly prominent C=O stretching band, likely due to the absence of the imprinting effect in the polymer structure. All the IR spectra confirmed the efficiency of the polymerization and imprinting processes.

### Surface area and porosity analysis

Brunauer–Emmett–Teller analysis (BET) was employed to estimate the surface area and the porosity of the synthesized MIPs and their corresponding NIP. The three polymers were subjected to surface cleaning and degassing by nitrogen flow at 100 °C for 4 h to eliminate adsorbed moisture. Then, the adsorption/desorption isotherms [Fig. S2†] was obtained by carrying out the measurements in liquid nitrogen at a temperature of −196°C. These isotherm measurements were used to calculate the specific surface area of the polymers [Table 1] *via* the BET equation.^58^ The adsorption isotherms were also utilized to estimate the average pore volumes and diameters through the non-local density functional theory.^59^ The presented results confirmed the high surface area and porosity of the MIPs over the NIP, ensuring the success of the imprinting process.

**Table 1:** Results of BET analysis and binding capacity calculations for the MIPs and their corresponding NIP

Polymer	Specific surface area (m^2^ g^−1^)	Pore volume (cm^3^ g^−1^)	Average pore diameter (nm)	Binding capacity *Q* (mmol g^−1^)	Imprinting factor (IF)	Selectivity evaluation by *Q* (mmol g^−1^)
DON-MIP	237.53	0.25	4.46	0.047	2	0.0211
MEM-MIP	248.76	0.28	4.61	0.03441	1.43	0.027
NIP	200.95	0.22	4.41	DON: 0.0235	NA	—
				MEM: 0.024		

### Binding capacity

The ability of the leached MIPs to rebind with their templates defines their functionality as recognition ionophores. The reported HPLC methods of DON^44^ and MEM^45^ were used to assess the binding capacity of the three polymers toward the studied drugs. DON binding capacity was assessed by adding 20 mg of DON-MIP or NIP into a 10-mL volumetric flask containing 0.1 mM of DON dissolved in phosphate buffer pH 5.5. The contents were mixed and stirred for 2 h as an incubation time, then they were centrifuged at 4000 rpm for 15 min. The clear supernatants were injected into the HPLC column through a syringe fitted with a 0.22 μm syringe filter to proceed with the HPLC analysis. The process was repeated for MEM as stepped using 0.069 mM of MEM solution instead.

The binding capacity (*Q*) of each polymer was calculated using the following equation:^60^
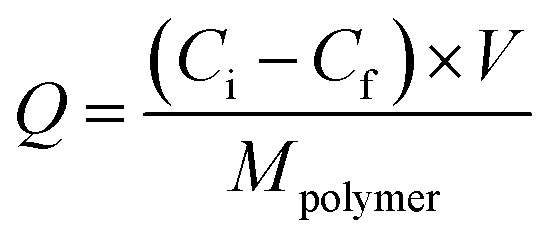
where *C*_i_ and *C*_f_ are the initial and final remaining drug concentrations in mM, *V* is the prepared solution volume in L, and *M*_polymer_ is the added mass of the polymer in g.

The binding capacities were used to calculate the imprinting factor (IF) of the MIPs through dividing the *Q* value of each MIP by that of its respective NIP. The IF values presented in Table 1 are higher than 1, which confirm the prevalence of the specific recognition in the MIPs over the non-specific one in the NIP.

Furthermore, the selectivity of the MIPs was evaluated by calculating the *Q* values in the co-formulated interfering drug rather than the corresponding drug, preferably in the same concentration. However, DON-MIP was incubated in 0.069 mM MEM solution rather than 0.1 mM owing to the limit of the linearity range of HPLC method for MEM. Therefore, the incubation time was increased to 3 h to compensate for the concentration differences. The *Q* values of the selectivity evaluation [Table 1] ensured the binding selectivity of each MIP towards its respective drug.

### Size distribution of graphene nanoplatelets

DLS analysis in methanol showed a zeta-average dynamic diameter of 2989 nm with a polydispersity index (PDI) of 0.539 [Fig. S3†], indicating relatively uniform dimensions and good dispersion. THF was used in the preparation of the transducer layer due to its ability to disperse the hydrophobic nanoplatelets, in addition to rapid evaporation without aggregation diving a uniform layer. However, methanol was selected for size analysis to be compatible with the cuvette and the instrument.

### Performance of the studied sensors

**Fig. 3:**
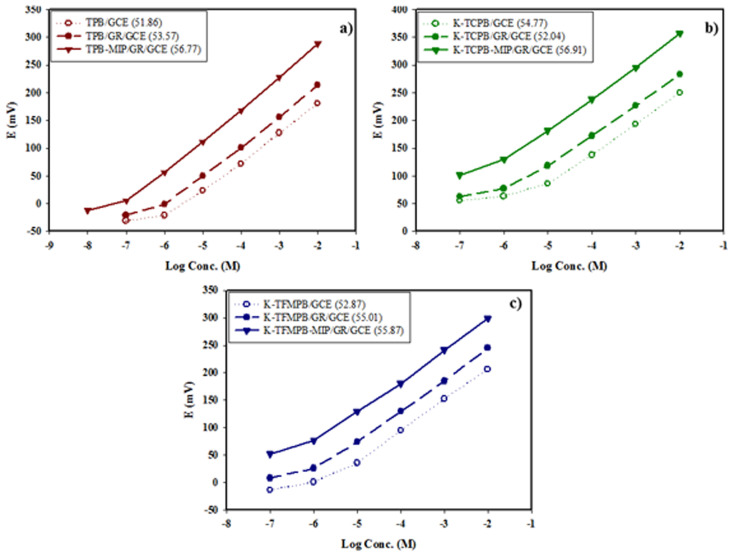
Potential profile in mV *vs.* log concentration of: (a) DON by TPB sensors: MIP/GR/GCE (1.0 × 10^−7^ to 1.0 × 10^−2^ M), GR/GCE (1.0 × 10^−6^ to 1.0 × 10^−2^ M) and unmodified GCE (1.0 × 10^−6^ to 1.0 × 10^−2^ M), (b) DON by K-TCPB sensors: MIP/GR/GCE (1.0 × 10^−6^ to 1.0 × 10^−2^ M), GR/GCE (1.0 × 10^−6^ to 1.0 × 10^−2^ M) and unmodified GCE (1.0 × 10^−5^ to 1.0 × 10^−2^ M), (c) MEM by K-TFMPB sensors: MIP/GR/GCE (1.0 × 10^−6^ to 1.0 × 10^−2^ M), GR/GCE (1.0 × 10^−6^ to 1.0 × 10^−2^ M) and unmodified GCE (1.0 × 10^−6^ to 1.0 × 10^−2^ M), all along with their corresponding slopes.

The electrochemical performance of the studied sensors was evaluated according to IUPAC recommendations.^61,62^ The GR deposit was employed as a water-repellent transducer between ISMs and GCEs, and the sensors' characteristics are presented in Table 2. Fig. 3 illustrates the potential profile in mV *versus* log concentrations of either DON or MEM for the proposed sensors. The GR-modified sensors exhibited higher Nernstian slopes, lower LOD values, and lower response time than the unmodified sensors. The increased surface area of graphene nanoplatelets and their enhanced capacitance improved the performance characteristics of the GR-modified sensors. Afterward, MIPs were added to the ISMs composition for selectivity enhancement. The added mass of the MIPs was assessed regarding the effect on sensors' performance and selectivity.^57,63^ Amounts of 1 mg, 2 mg, 5 mg, 10 mg, 15 mg and 20 mg of each corresponding MIP were added separately to TPB-ISM and K-TCPB-ISM for DON and K-TFMPB-ISM for MEM. Increasing the amounts up to 10 mg gradually improved the slope, performance, precision, and selectivity of the proposed sensors. No significant improvements were noticed with the 15 mg amount. However, non-uniform dispersion of the MIPs particles and dense ISM surfaces were observed with the 20 mg amount accompanied by a decrease in sensors' slopes and response potential. Therefore, 10 mg amount was employed to complete the study. The noted improvements in the performance and selectivity of the proposed sensors, as well as the remarkably lowered LOD values, are likely due to the specific binding sites in the synthesized MIPs that enhanced the recognition of the corresponding drug. It was also noted that DON-MIP highly improved the slope and detection limit compared with the MIP-free sensors and even with the improvement obtained with the MEM-MIP sensor. Each MIP-sensor was connected and evaluated individually using the same reference electrode, without simultaneous connection of multiples sensors.

**Table 2:** Electrochemical responses of the proposed MIP/GR-modified sensors compared with MIP free-GR-modified and unmodified sensors

Parameter	DON						MEM		
	TPB/GCE			K-TCPB/GCE			K-TFMPB/GCE		
	MIP/GR	GR	Plain GCE	MIP/GR	GR	Plain GCE	MIP/GR	GR	Plain GCE
Slope^a^ (mV per decade)	56.77	53.57	51.86	56.91	52.04	54.77	55.87	55.01	52.87
Intercept (mV)	398.34	317.76	279.76	467.86	383.54	358.16	408.68	351.64	309.4
Correlation coefficient (*r*)	0.99955	0.9996	0.999	0.9995	0.9984	0.9997	0.9994	0.9992	0.9966
Concentration range (M)	10^−7^ to 10^−2^	10^−6^ to 10^−2^	10^−6^ to 10^−2^	10^−6^ to 10^−2^	10^−6^ to 10^−2^	10^−5^ to 10^−2^	10^−6^ to 10^−2^	10^−6^ to 10^−2^	10^−6^ to 10^−2^
LOD^b^ (M)	5.01 × 10^−8^	3.98 × 10^−7^	5.6 × 10^−7^	4.47 × 10^−7^	7.08 × 10^−7^	3.98 × 10^−6^	2.24 × 10^−7^	4.47 × 10^−7^	7.08 × 10^−7^
Stability (days)	45	45	35	50	50	40	50	50	40
Response time (s)	5–10	5–10	15–20	5–10	5–10	15–20	5–10	5–10	15–20
Working pH range	3–6.5	3–6.5	3–6.5	3–6.5	3–6.5	3–6.5	4–8.5	4–8.5	4–8.5
Accuracy (mean ± SD)^c^	100.66 ± 1.18	100.98 ± 1.48	100.20 ± 2.01	99.81 ± 1.04	99.45 ± 1.74	100.44 ± 1.31	101.24 ± 1.17	100.08 ± 1.67	99.28 ± 1.79

**Precision**									
(RSD%)^d^	0.92	1.48	1.78	1.22	1.52	1.21	1.18	1.56	1.63
(RSD%)^e^	1.56	1.78	1.87	1.68	1.79	1.56	1.60	1.77	1.88
Reproducibility (RSD%)^f^	1.89	—	—	1.70	—	—	1.83	—	—

**Dosage form (recovery% ± SD)** a									
DON/MEM 10/14 mg per tab	99.15 ± 0.88	—	—	100.83 ± 1.11	—	—	100.69 ± 1.37	—	—
DON/MEM 10/28 mg per tab	100.62 ± 1.05	—	—	101.56 ± 1.54	—	—	101.46 ± 1.14	—	—
Plasma (recovery% ± SD)^a^	96.89 ± 1.35	—	—	97.90 ± 1.76	—	—	95.91 ± 1.37	—	—

a Average of three determinations.

b Limit of detection (measured by interception of the extrapolated arms of potential profiles figures.

c Mean ± SD of three concentrations measured in triplicates.

d Intraday precision or repeatability (average of three different concentrations measured in triplicates in the same day).

e Interday precision or intermediate precision (average of three different concentrations measured in triplicates for three days).

f Average of three different concentration with three batches of the sensors.

The response time is defined as the duration from the moment the reference electrode and proposed sensors contact the sample solution until a steady response (±1 mV) is achieved. The response time for unmodified sensors was about 15 s to 20 s, while for GR-modified sensors and MIP/GR-modified sensors, it was about 5 s to 10 s. This improvement suggests absence of water layer beneath the sensing membrane, which otherwise acts as a diffusion barrier and electrolytes' reservoir, delaying the signal stabilization.

The lifetime of GR-modified and MIP/GR-modified sensors was found to be between 45 to 50 days, as they retained their potential stability and Nernstian response throughout this period.

### Influence of GR as a transducer layer on GCE

As previously discussed, incorporating GR as a transducer layer significantly improved and stabilized the potential response of the modified sensors. This effect is attributed to the GR layer's ability to hinder the formation of a water layer between the GCE surface and the ISM, in addition to enhancing the charge transfer between the sensing layers. These influences became even more significant in MIP-based sensors, where GR supports the sensing membrane physically and electronically, ensuring efficient signal transduction by facilitating signal transfer between the MIPs as recognition elements and the electrode surface.

#### Water layer test

**Fig. 4:**
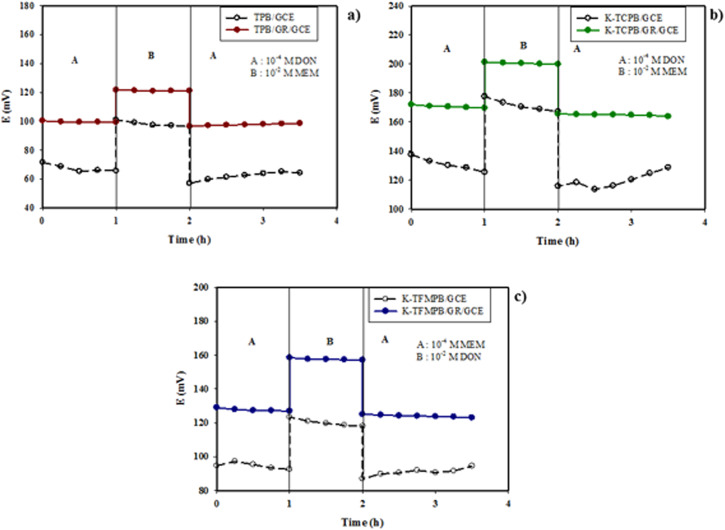
Water layer test of: (a) DON by TPB sensors, (b) DON by K-TCPB sensors, (c) MEM by K-TFMPB sensors, using GR-modified and unmodified sensors.

The water layer test was performed to assess the potential stability by exposing the unmodified and GR-modified sensors to a higher concentration of the interfering drug. The potential of each sensor was first measured in 1 × 10^−4^ M solution of its corresponding drug, followed by the exposure to 1 × 10^−2^ M solution of the interfering drug, and finally returned to its main drug solution. Significant potential drifts were observed in the unmodified sensors as a result of ion fluctuation in the water layer. Conversely, the stable potentials observed in the GR-modified sensors indicated that the GR nanoplatelets inhibited the development of a water layer below the ISM [Fig. 4].

#### Impedance study

An Electrochemical Impedance Spectroscopy (EIS) analysis was conducted to assess the graphene transducer layer. The Bode graphs for both unmodified and GR-modified sensors [Fig. S4†] showed a reduction in transfer resistance in the GR-modified sensors, which enhances charge transfer.

### Assessing sensors' performance at various pH values

The influence of solution pH on the potential responsiveness of MIP sensors was investigated over a pH range of 2–11 to determine the optimal settings. The response of 1 × 10^−4^ M and 1 × 10^−3^ M of DON and MEM solutions was measured at different pH levels and the emf responses were plotted within the specified pH range [Fig. S5†]. The response of DON's sensors remained nearly uniform within a pH range of 3.0 to 6.5; other studied pH values showed no constant region. pH higher than 8.0 caused drug precipitation. The proposed sensor for MEM showed a relative constant potential response at a pH region of 4.0 to 8.5; lower and higher pH values showed no constant region as well. Potential responses in lower pH values were slightly higher than the constant region, likely due to a high concentration of hydrogen ions, while the lower responses obtained in higher pH values were mainly attributed to the decrease in MEM cationic form with increasing pH value. Therefore, a pH value of 5.5 was used for simultaneous analysis of both drugs.

### Selectivity of the sensors

**Fig. 5:**
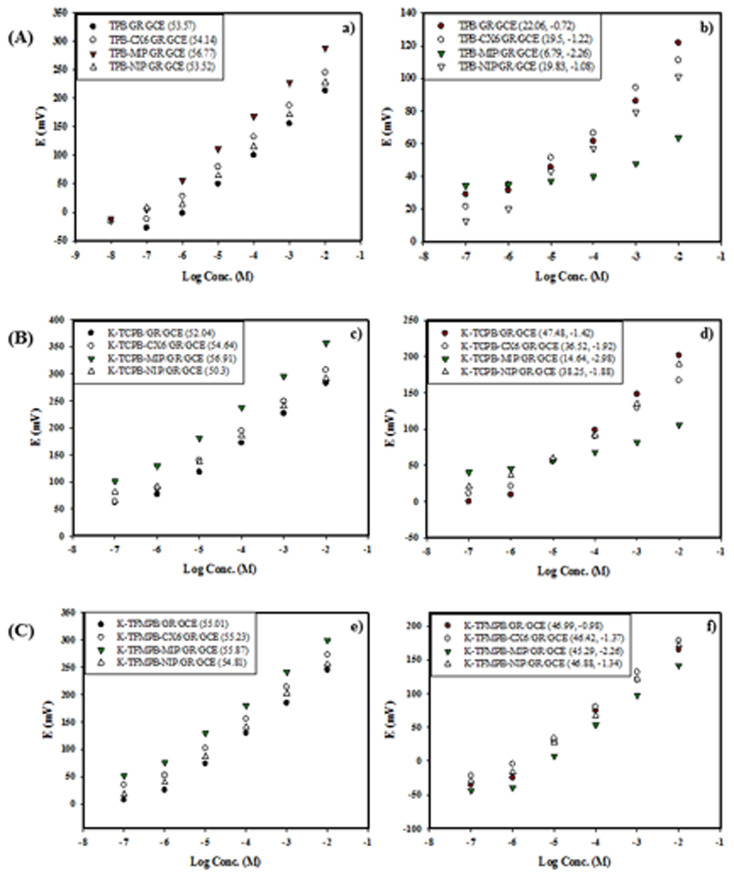
Response of various sensors of: (A) DON-TPB as function of log concentration of (a) DON and (b) MEM, (B) DON-TCPB as function of log concentration of (c) DON and (d) MEM, (C) MEM-TFMPB as function of log concentration of (e) MEM and (f) DON, where values between parentheses represent the slope in (a, c and e) figures, and slope and (log *K*^pot^_drug,I_) in (b, d and f) figures.

The selectivity and specificity of the proposed sensors were evaluated by measuring the potential of 1 × 10^−4^ M solution of the target drug alongside an equivalent concentration of the interfering ions including co-formulated drug, routinely used additives and cations' solutions] utilizing separate solution method (SSM).^64^ The unbiased potentiometric selectivity coefficient (*K*^pot^_primaryion,interferent_) was employed to measure the selectivity of the MIP/GR sensors (Table 3), calculated according to the following equation: log(*K*^pot^_primaryion,interferent_) = −(*E*_1_ − *E*_2_)/*S*, where *E*_1_ represents the measured potential of 1 × 10^−4^ M of the main drug, *E*_2_ denotes the measured potential of the interfering ion at the identical concentration and *S* signifies the slope of the sensor. While this equation was formulated mainly for ionic interferents, it was also used for neutral compounds to give a qualitative indication of their influence, as they may be present in pharmaceutical or biological matrices. Firstly, MIP/GR-modified sensors for each drug were compared with the matching GR-modified sensors, NIP/GR-modified sensors, in addition to CX6/GR-modified sensors as an example of ionophore-decorated sensors.^65^ The comparison was based on performance with the target drug [Fig. 5a, c and e] as well as selectivity towards the interfering drug [Fig. 5b, d and f] through the construction of calibration curves in each case to obtain the slope and calculate the selectivity coefficient in the selectivity study.

**Table 3:** Selectivity coefficient (log *K*^pot^_drug,I_) of the proposed sensors using separate solution method

	Log *K*^pot^_DON,I_		Log *K*^pot^_MEM,I_
Interferent (I)	TPB-MIP/GR/GCE	K-TCPB-MIP/GR/GCE	K-TFMPB-MIP/GR/GCE
MEM	−2.26	−2.98	—
DON	—	—	−2.26
Na^+^	−3.06	−3.75	−3.16
K^+^	−3.15	−3.70	−3.15
Ca^2+^	−3.13	−3.79	−3.26
Mg^2+^	−2.86	−3.50	−3.27
Glucose	−3.18	−3.88	−3.31
Lactose	−3.31	−3.59	−3.35
Starch	−3.46	−3.97	−3.40

Although the incorporation of CX6 as an ionophore improved the performance and selectivity of the sensors, MIP-modified sensors demonstrated significantly enhanced slopes, lower detection limits, and improved selectivity (by approximately two orders of magnitude), confirming the superiority of the MIPs for the analysis of the drug mixtures. The results revealed that DON-MIP not only improved the selectivity for DON over MEM but also substantially diminished the sensors' response to MEM. That was evidenced by the reduced slopes and the nearly flat calibration curves. This behavior suggested that specific binding to DON dominated over non-specific binding sites in DON-MIP. It was important to note that DON had a lower mole fraction in the dosage forms, making the efficient DON-MIP highly beneficial for this analysis. Furthermore, the selectivity of the MIP/GR-modified sensors against the inorganic cations and the commonly used additives was examined. The calculated selectivity coefficients revealed no observed interference from these substances, likely due to the differences in ionic size and lipophilicity of the studied inorganic cations and the absence of ionic charges in the studied additives.

### Applications to pharmaceutical dosage forms and human plasma

The MIP/GR-modified sensors were used to determine corresponding drugs in their formulated capsules Mixmazil® and extended to human plasma to prove the sensors' selectivity in actual samples (Table 2). Results indicated absence of interference between the co-formulated drugs and the excipients in the formulation. Also, DON and MEM presented satisfactory results upon analysis in spiked human plasma after extraction. Consequently, the sensors can determine DON and MEM within their formulated capsules without necessitating prior separation or treatment and in human plasma after extraction and protein precipitation with methanol due to the high binding of DON to the plasma proteins.

### Statistical evaluation of the proposed method

To estimate the sensors' validity, their results were statistically compared to the corresponding reported method for each drug.^44,45^ The computed Student's *t*-test and F-test results indicated no significant difference between the proposed MIP/GR-modified sensors and the corresponding reported methods (Table S1†). Additionally, the proposed method enables the simultaneous analysis of both drugs in rapid, cost-effective, and sustainable analytical procedures.

### Green profile and whiteness assessment metrics

The advancement of eco-friendly techniques has become increasingly prevalent in analytical science.^1,34,66–69^ To judge the greenness and sustainability of the suggested approach, several metric tools were employed, including AGREE, the whiteness tool (WAC), and MoGAPI (Table 4).

**Table 4:** Greenness and whiteness assessment comparison between the proposed and reported methods

	Proposed method	DON reported	MEM reported
AGREE	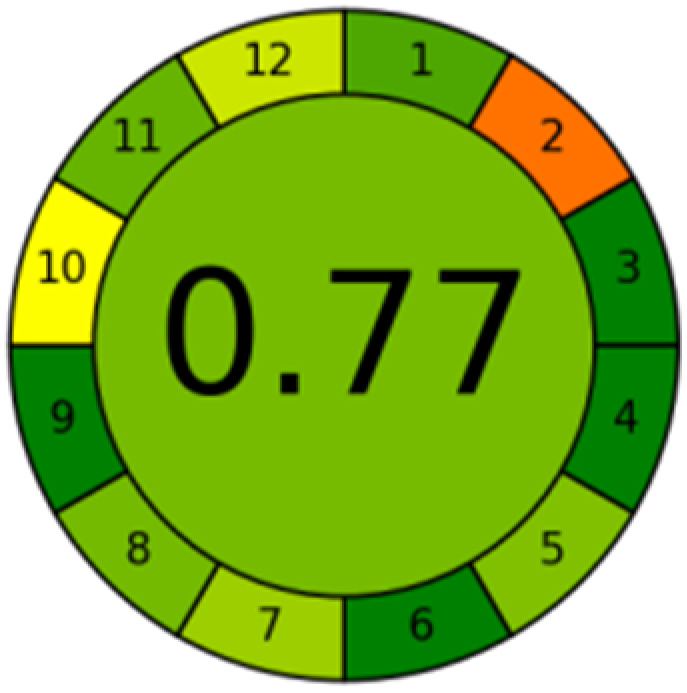	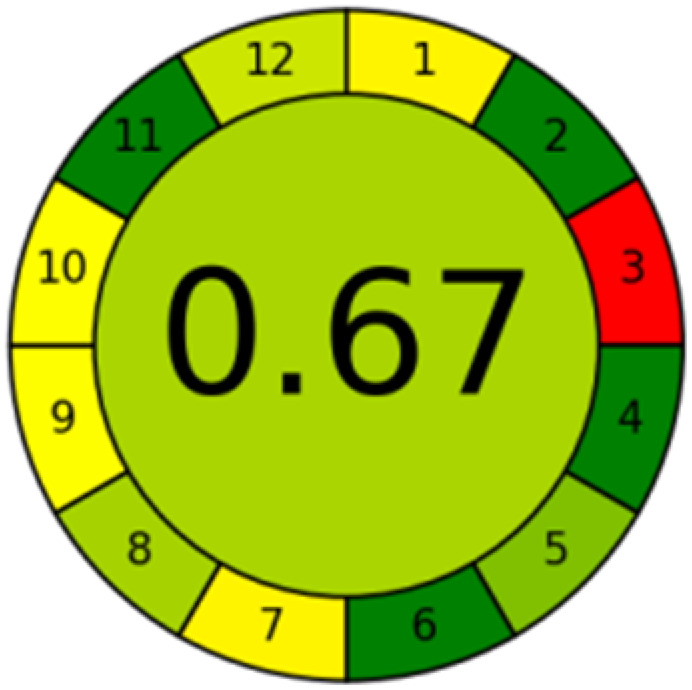	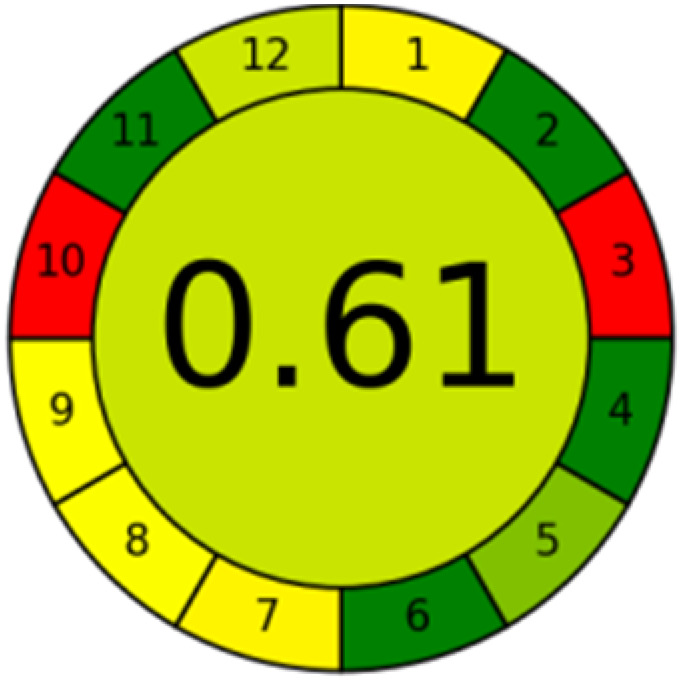
WAC	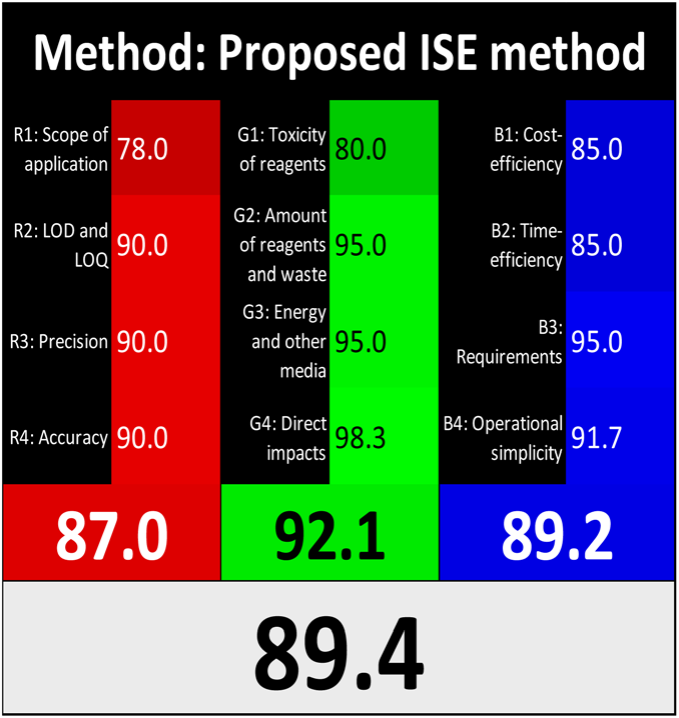	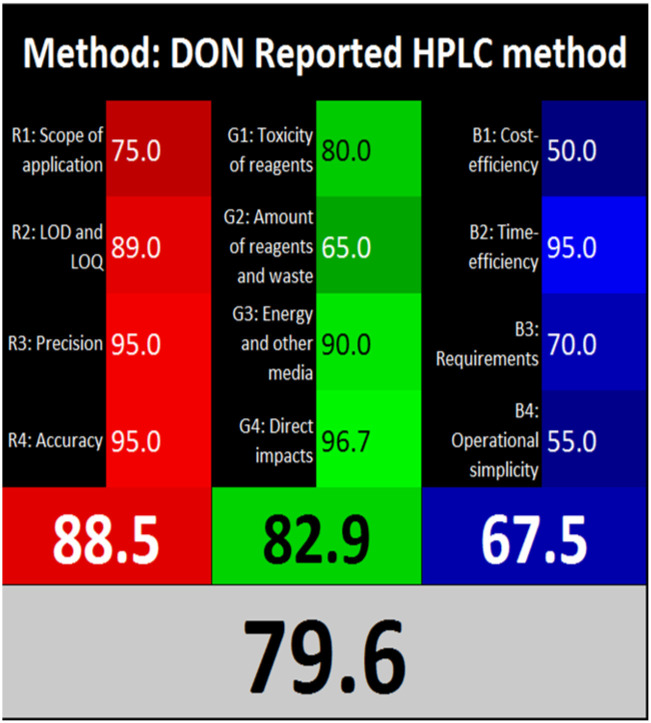	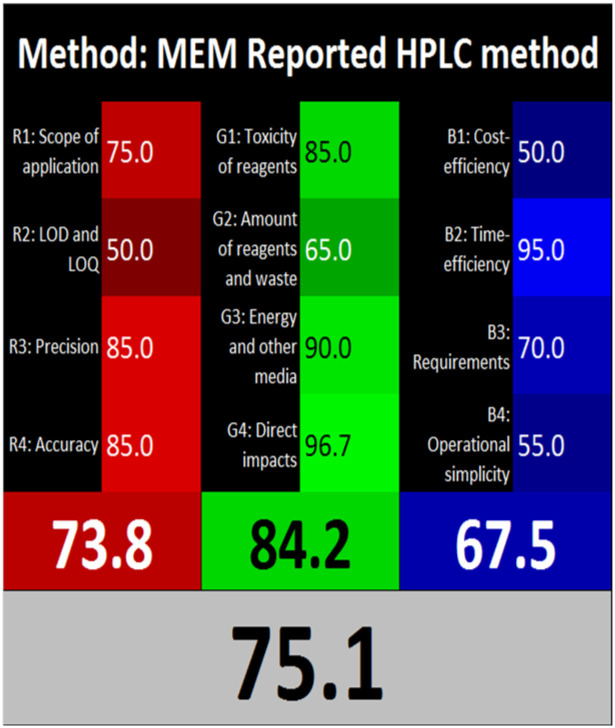
MoGAPI	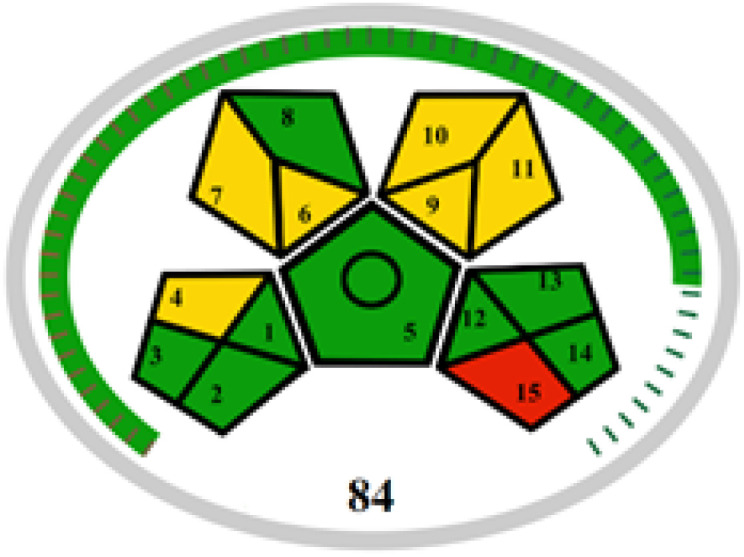	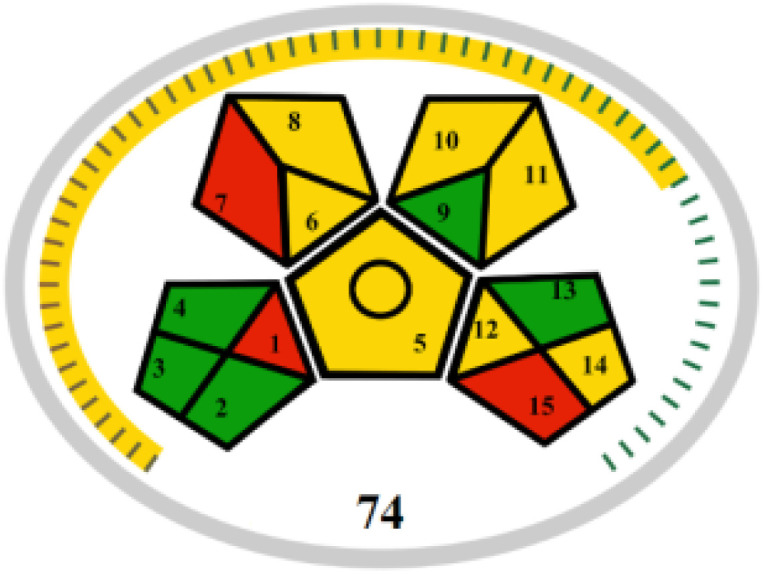	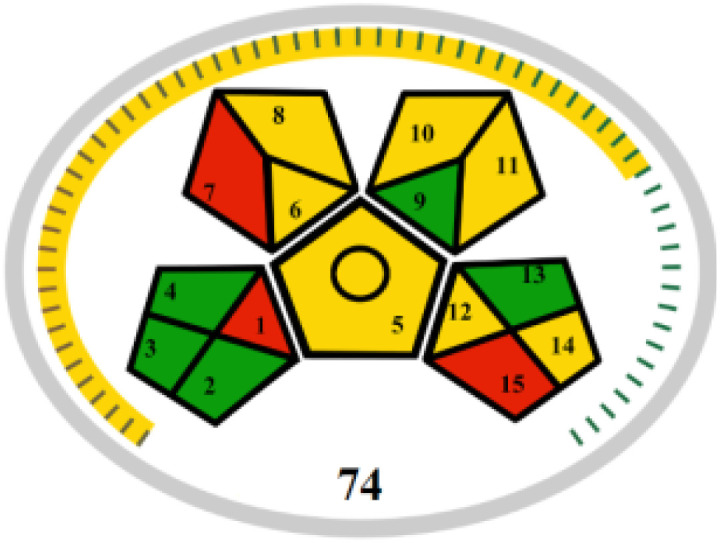

The AGREE tool uses a circle like clock pictogram to give both a quantitative and visual depiction of a method's adherence to the 12 codes of green analytical chemistry, assigning a score between 0 and 1. A score approaching 1 indicates a greener method.^41^ The proposed method achieved a score of 0.77, compared with 0.67 and 0.61 for DON and MEM reported methods, respectively.

The whiteness tool (WAC) evaluates the analytical efficacy, environmental safety, and practicality of the method through a Red-Green-Blue model. This model assesses sustainability and produces a color shade ranging from black to white based on three factors: red for analytical performance, green for safety and environmental impact, and blue for practicality and economic considerations. These factors are quantitatively combined to generate a ranking from 0 to 100, where black represents the least favorable method and white represents the most favorable one.^42^ The proposed method achieved a score of 89.4, surpassing the DON and MEM reported methods' scores of 79.6 and 75.1, respectively.

The MoGAPI tool utilizes a segmented pictogram with color codes red, yellow, and green, indicating high, medium, and low environmental hazards, respectively. Moreover, MoGAPI offers online software that calculates the total score and provides segment numbers for a more straightforward interpretation of their significance. Each step of the analytical process is assessed individually for its ecological impact.^43^ For the proposed method, the MoGAPI pictogram primarily displayed green, suggesting negligible ecological influence with some yellow areas and a red segment due to lack of waste treatment. The total score obtained for the reported method is 84, and both of the reported methods scored 74, confirming the greater greenness of the proposed method.

Overall, the suggested method demonstrated enhanced green and white profiles relative to the evaluated reported methods.

## Conclusion

This work demonstrates MIPs-doped sensors for the direct determination of two positively charged drugs, DON and MEM, combined in pharmaceutical capsules to manage AD. Selective sensing is based on the imprinted cavities in the MIPs that act as synthetic receptors for their corresponding drugs through specific binding and recognition. The effectiveness of this merit was assessed for each MIP using rebinding studies by FT-IR analysis in comparison with leached/template-free MIPs, in addition to rebinding capacity measurements. The potentiometric measurements demonstrated the significant influence of the MIPs on the performance of the modified sensors regarding detection limits, slopes, and selectivity towards the interfering drug compared with MIP-free sensors. DON-MIP exhibited a notable selectivity impact by improving the detection limits and selectivity toward DON over MEM, in addition to almost flattening the sensing of MEM. The sensors were also modified with GR nanoplatelets as a transducer layer. The presence of GR produced rapid, stable, and reproducible responses due to prevention of water layer formation beneath the ISM. The sensors' sensitivity and selectivity enabled the simultaneous determination of the studied drugs in their combined formulations and spiked human plasma samples. The study suggests the application of the proposed method as a rapid, sustainable, intuitive, and equipment-less alternative analytical technique.

## Data availability

The data are available from the corresponding author on reasonable request.

## Author contributions

Eman M. Moaaz: conceptualization, methodology, formal analysis, visualization, investigation, validation, writing – original draft. Ahmed S. Fayed: methodology, resources, resources, writing – review & editing, supervision. Ezzat M. Abdel-Moety: writing – review & editing, supervision. Mamdouh R. Rezk: conceptualization, resources, writing – review & editing, supervision.

## Conflicts of interest

The authors have declared no conflict of interest.

## Supplementary Material

RA-015-D5RA02850G-s001
