# Supplementary material for: Innovative sensors with selectivity enhancement by molecularly imprinted polymers for the concurrent quantification of donepezil and memantine
Source: RSC Adv. 2025 Jun 3;15(23):18475–89. doi: 10.1039/d5ra02850g (PMC12132094; doi:10.1039/d5ra02850g)
Supplement: RA-015-D5RA02850G-s001 [file RA-015-D5RA02850G-s001.pdf]

Supplementary Information (SI) for RSC Advances.
This journal is © The Royal Society of Chemistry 2025

# Innovative sensors with selectivity enhancement by molecularly imprinted polymers for the concurrent quantification of donepezil and memantine

Eman M. Moaaz*, Ahmed S. Fayed, Ezzat M. Abdel-Moety, Mamdouh R. Rezk

*Pharmaceutical Analytical Chemistry Department, Faculty of Pharmacy, Cairo University, Kasr El-Aini Street, ET-11562 Cairo, Egypt*

*** Corresponding author email: eman.moaaz@pharma.cu.edu.eg**

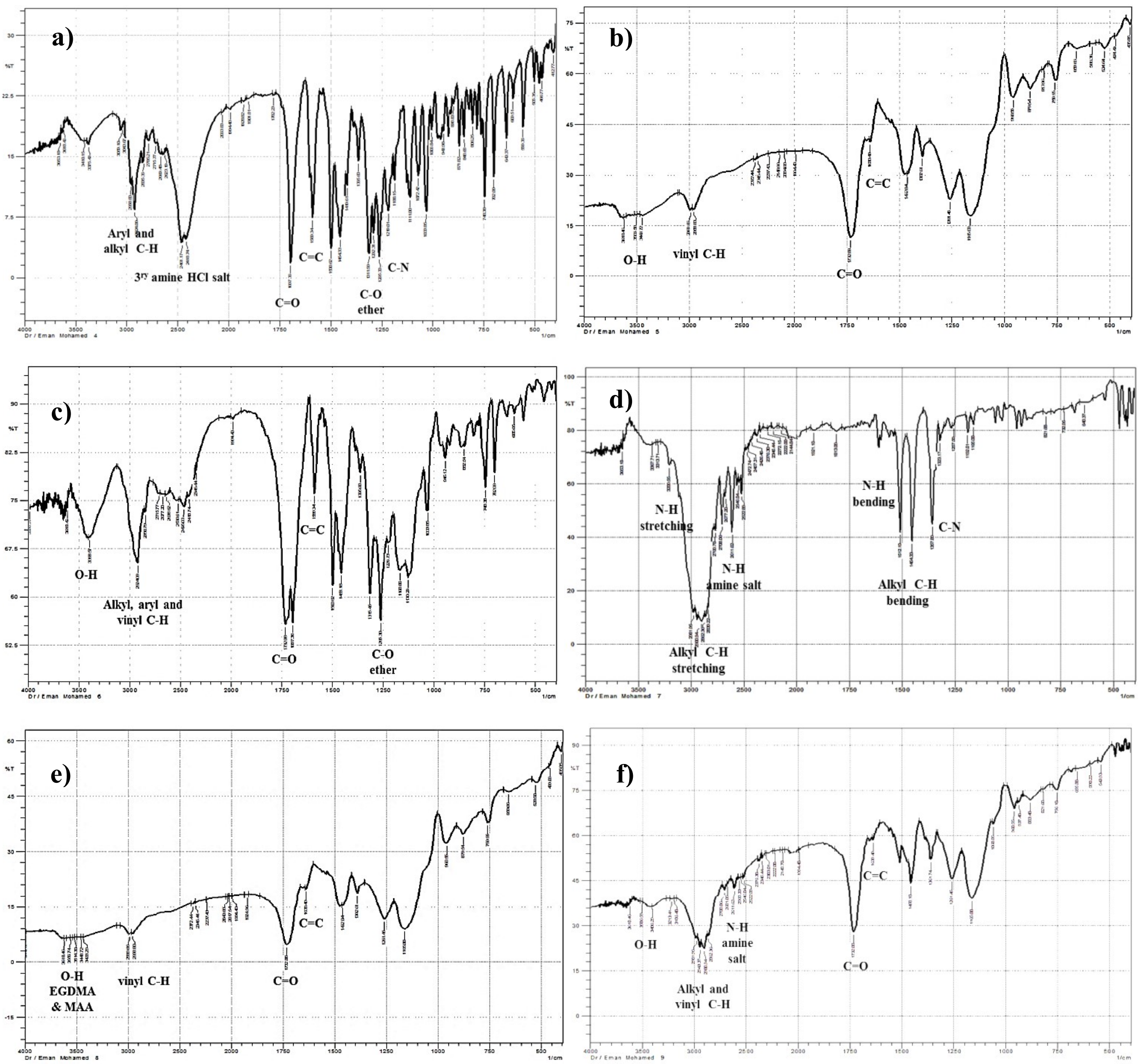

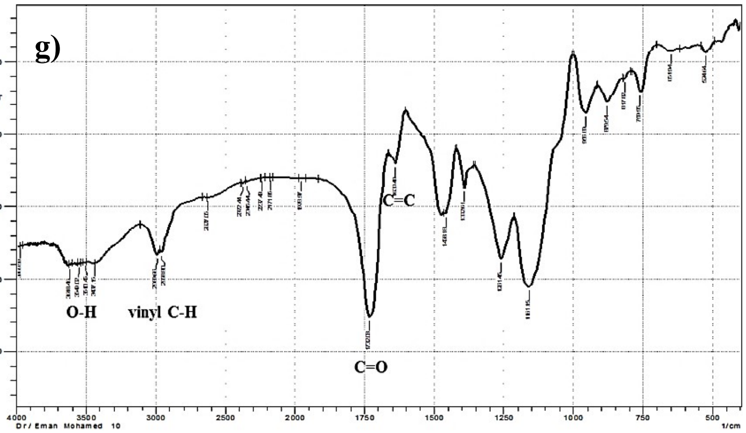


**Fig. S1.** FT-IR spectra of **a)** DON, **b)** leached DON-MIP, **c)** un-leached DON-MIP, **d)** MEM, **e)** leached MEM-MIP, **f)** un-leached MEM-MIP, **g)** NIP.

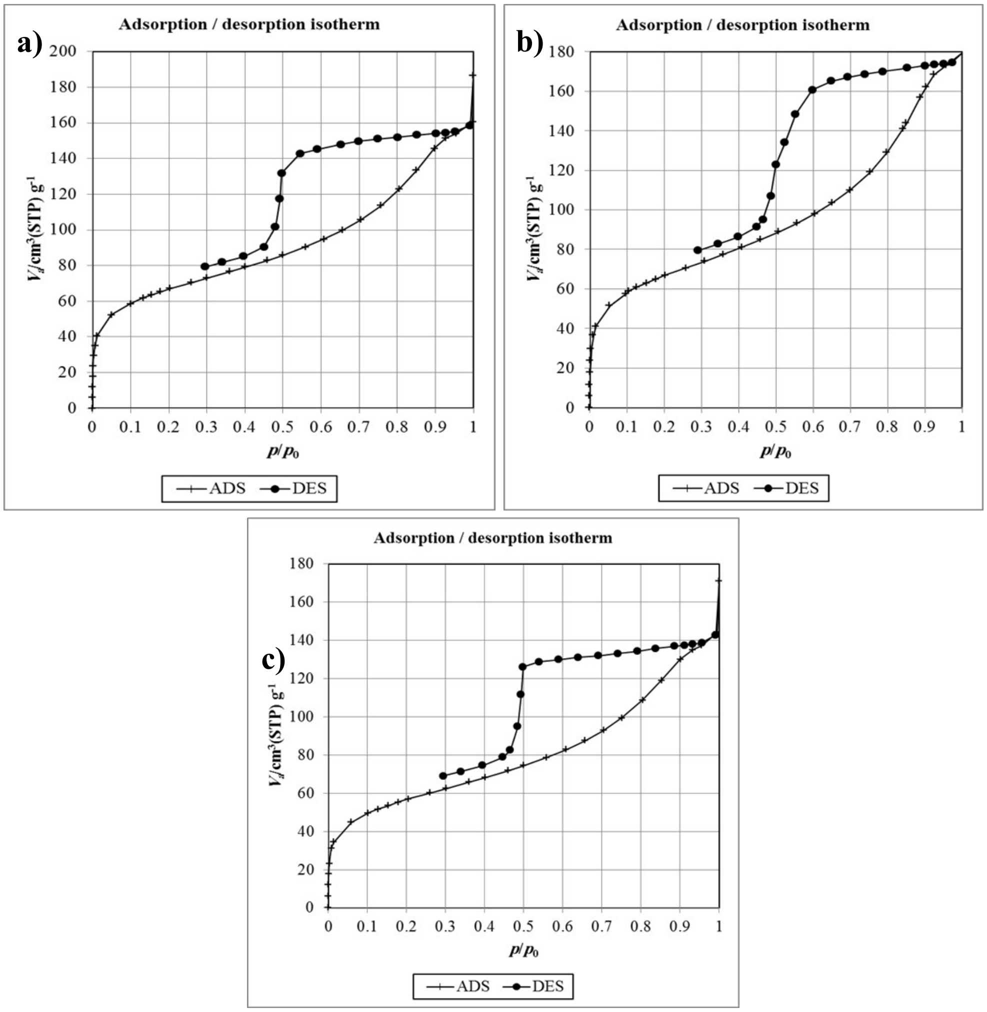


**Fig. S2.** Nitrogen adsorption/desorption isotherm of **a)** DON-MIP, **b)** MEM-MIP, **c)** NIP

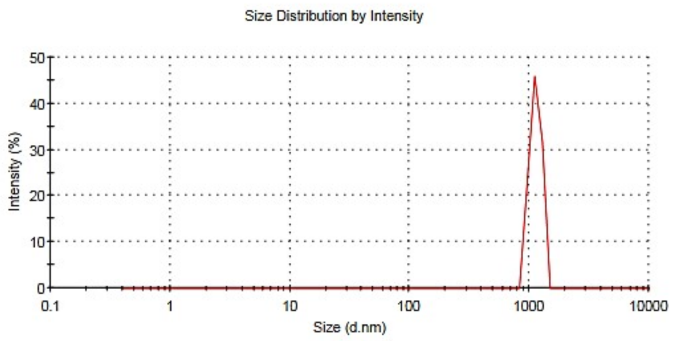


**Fig. S3.** DLS size distribution graph of graphene nanoplatelets dispersed in methanol.

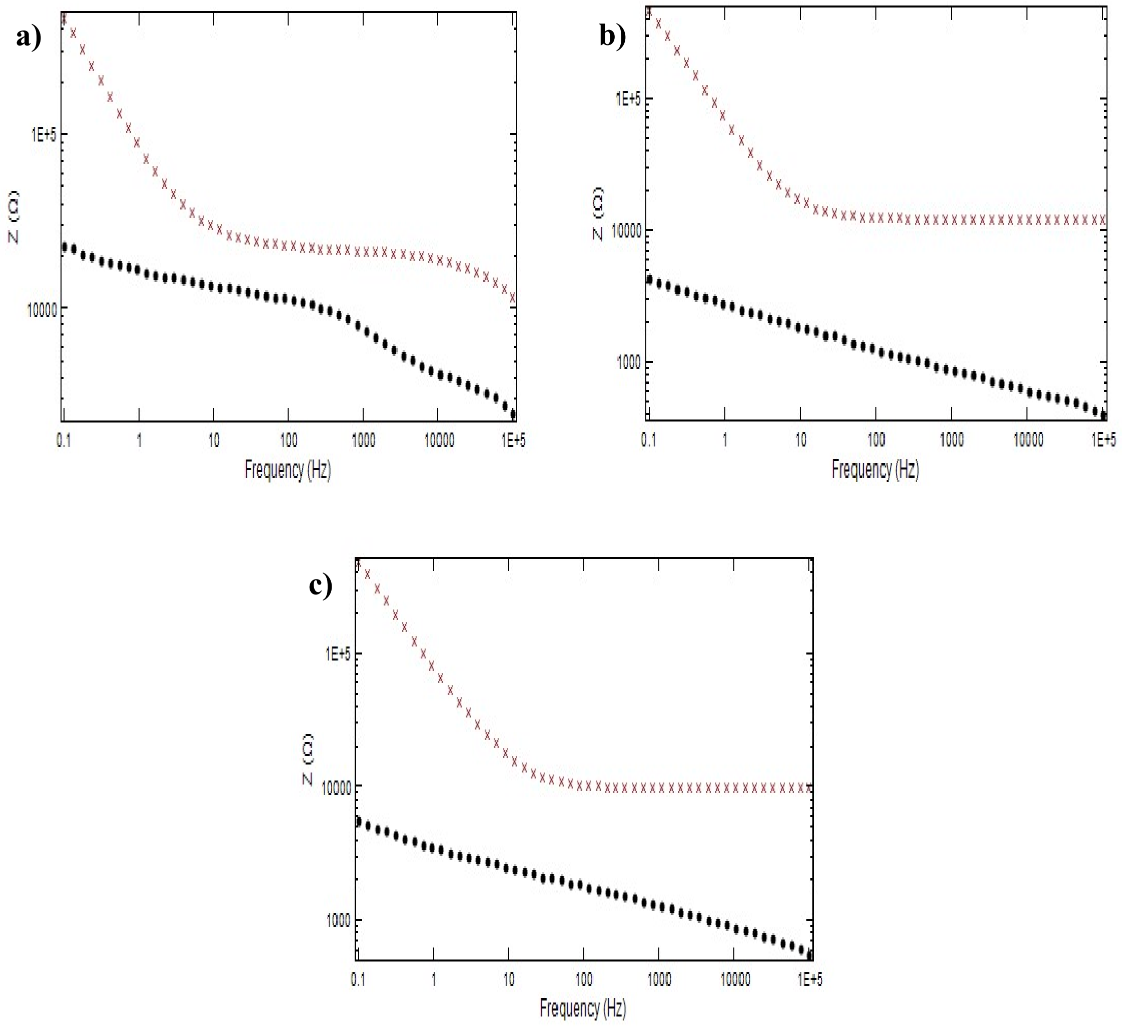


**Fig. S4.** Bode plots of GR-modified (**….**) and unmodified (xxxx) GCEs of: **a)** DON-TPB sensors, **b)** DON-K-TCPB sensors, **c)** MEM-K-TFMPB sensors.

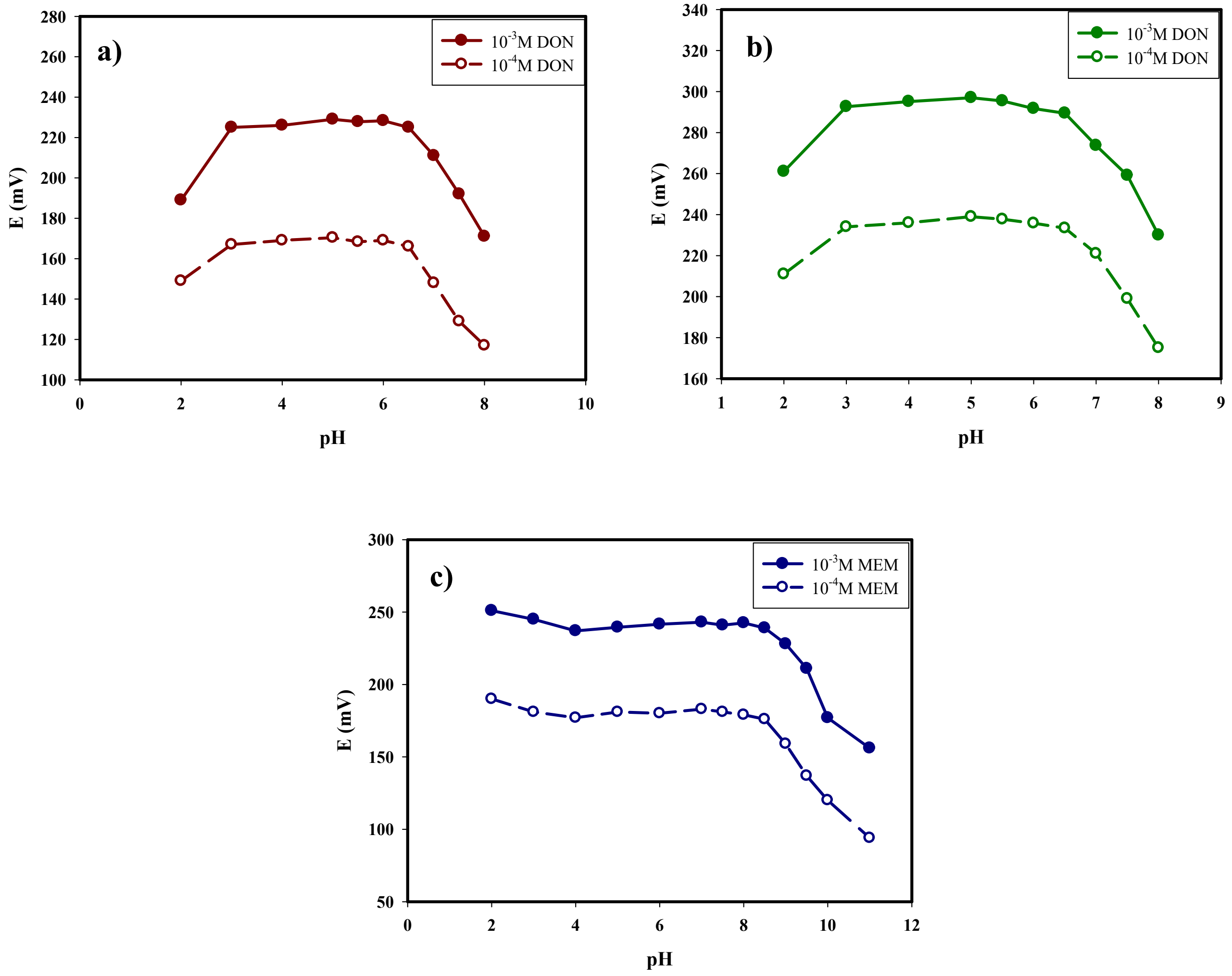


**Fig. S5.** Effect of pH on the *emf* of the proposed MIP/GR/GCE sensors: **a)** DON-TPB sensor, **b)** DON-K-TCPB sensor, **c)** MEM-K-TFMPB sensor.

**Table S1.** Statistical comparison of the results obtained by the proposed sensors and the reported methods

| | DON | | | MEM | |
|---|---|---|---|---|---|
| **Value** | **TPB-MIP/GR/GCE** | **K-TCPB-MIP/GR/GCE** | **Reported method[a]** | **K-TFMPB-MIP/GR/GCE** | **Reported method[b]** |
| **Mean** | 99.80 | 99.84 | 100.01 | 99.86 | 99.95 |
| **SD** | 1.64 | 1.64 | 1.45 | 1.54 | 1.80 |
| **%RSD** | 1.64 | 1.64 | 1.45 | 1.54 | 1.80 |
| **Variance** | 2.690 | 2.690 | 2.103 | 2.372 | 3.240 |
| **N** | 5 | 5 | 5 | 5 | 5 |
| **t-test[c] (2.306)** | 0.215 | 0.174 | | 0.085 | |
| **F Value[c] (6.39)** | 1.279 | 1.279 | | 1.366 | |

[a] HPLC method ($C_{18}$) with mobile phase composed of methanol: water (pH 4 adjusted with orthophosphoric acid) in ratio of 60: 40. The flow rate was adjusted at 1.0 mL/min and UV detection at 230.0 nm.

[b] HPLC method ($C_8$) with mobile phase composed of acetonitrile: 0.1% orthophosphoric acid in ratio of 1: 99. The flow rate was adjusted at 1.0 mL/min and UV detection at 190.0 nm.

[c] The values in the parenthesis are the corresponding theoretical values of t and F at P = 0.05.
